# Mortality Among Professional American-Style Football Players and Professional American Baseball Players

**DOI:** 10.1001/jamanetworkopen.2019.4223

**Published:** 2019-05-24

**Authors:** Vy T. Nguyen, Ross D. Zafonte, Jarvis T. Chen, Kalé Z. Kponee-Shovein, Sabrina Paganoni, Alvaro Pascual-Leone, Frank E. Speizer, Aaron L. Baggish, Herman A. Taylor, Lee M. Nadler, Theodore K. Courtney, Ann Connor, Marc G. Weisskopf

**Affiliations:** 1 Department of Environmental Health, Harvard T.H. Chan School of Public Health, Boston, Massachusetts; 2 Department of Physical Medicine and Rehabilitation, Spaulding Rehabilitation Hospital, Massachusetts General Hospital, Brigham and Women's Hospital, and Harvard Medical School, Boston, Massachusetts; 3 Department of Social and Behavioral Sciences, Harvard T.H. Chan School of Public Health, Boston, Massachusetts; 4 Department of Epidemiology, Harvard T.H. Chan School of Public Health, Boston, Massachusetts; 5 Berenson-Allen Center for Noninvasive Brain Stimulation, Division of Cognitive Neurology, Department of Neurology, Beth Israel Deaconess Medical Center, Harvard Medical School, Boston, Massachusetts; 6 Channing Division of Network Medicine, Brigham and Women’s Hospital, Harvard Medical School, Boston, Massachusetts; 7 Cardiovascular Performance Program, Massachusetts General Hospital, Boston; 8 Cardiovascular Research Institute, School of Medicine, Morehouse University, Atlanta, Georgia; 9 Dana-Farber Cancer Institute, Harvard Medical School, Boston, Massachusetts; 10 Harvard Medical School, Boston, Massachusetts

## Abstract

**Importance:**

Studies of American-style football players have suggested lower overall mortality rates compared with general populations, but with possibly increased neurodegenerative mortality. However, comparisons with general populations can introduce bias. This study compared mortality between US National Football League (NFL) and US Major League Baseball (MLB) players, a more appropriate comparison group of professional athletes.

**Objective:**

To compare all-cause and cause-specific mortality between NFL and MLB players.

**Design, Setting, and Participants:**

In this retrospective cohort study, the setting was US mortality from January 1, 1979, through December 31, 2013. The dates of analysis were January 2016 to April 2019. Participants were 3419 NFL and 2708 MLB players with at least 5 playing seasons.

**Exposures:**

Participation in the NFL compared with the MLB.

**Main Outcomes and Measures:**

Vital status and causes of death from the National Death Index from 1979 through 2013 were obtained. Cox proportional hazards regression models using age as the timescale were used to calculate hazard ratios (HRs) and 95% CIs to examine all-cause and cause-specific mortality among NFL players compared with MLB players, adjusted for race and decade of birth.

**Results:**

By the end of follow-up, there were 517 deaths (mean [SD] age, 59.6 [13.2] years) in the NFL cohort and 431 deaths (mean [SD] age, 66.7 [12.3] years) in the MLB cohort. Cardiovascular and neurodegenerative conditions, respectively, were noted as underlying or contributing causes in 498 and 39 deaths in the NFL and 225 and 16 deaths in the MLB. Compared with MLB players, NFL players had significantly elevated rates of all-cause (HR, 1.26; 95% CI, 1.10-1.44), cardiovascular disease (HR, 2.40; 95% CI, 2.03-2.84), and neurodegenerative disease (HR, 2.99; 95% CI, 1.64-5.45) mortality. Comparing hypothetical populations of 1000 NFL and 1000 MLB players followed up to age 75 years, there would be an excess 21 all-cause deaths among NFL players, as well as 77 and 11 more deaths with underlying or contributing causes that included cardiovascular and neurodegenerative conditions, respectively.

**Conclusions and Relevance:**

This study found that NFL players had elevated all-cause, cardiovascular, and neurodegenerative mortality rates compared with MLB players, although the absolute number of excess neurodegenerative deaths was still small. Factors that vary across these sports (eg, body habitus and head trauma) as opposed to those common across sports (eg, physical activity) could underlie the differences.

## Key Points

**Question:**

What are the long-term health risks of National Football League (NFL) players compared with Major League Baseball (MLB) players, another group of elite athletes?

**Findings:**

In this cohort study of 3419 NFL and 2708 MLB players, NFL players had significantly higher mortality rates from all causes, cardiovascular diseases, and neurodegenerative diseases compared with MLB players.

**Meaning:**

This study found that NFL players had a higher rate of mortality than MLB players, which may be associated with aspects of playing in professional American-style football.

## Teaser

This cohort study compares all-cause and cause-specific mortality (including deaths associated with cardiovascular and neurodegenerative diseases) between US National Football League (NFL) and Major League Baseball (MLB) players.

## Introduction

There has been considerable interest, both in the scientific community and among the general public, in the lifelong health of American-style football players. Studies that have compared US National Football League (NFL) players with the general US population have found overall reduced mortality among NFL players. Such studies^1,2,3^ have been used in mainstream media to discredit claims that American-style football may have harmful consequences.^4,5^ In contrast to overall mortality, neurodegenerative disease mortality has been reported to be elevated among NFL players.^2^ The possibility that there may be an increased risk of neurodegenerative diseases among NFL players has received substantial attention after reports of neuropathology consistent with chronic traumatic encephalopathy in a number of professional American-style football players.^6,7^

However, comparisons of athletes with general populations can be biased. To become a professional athlete in any sport, one is typically healthier and fitter than people of a similar age in the general population, a bias known as the healthy worker hire effect.^8,9^ The bias is typically borne out with a reduced risk of overall mortality compared with the general population. Such comparisons have limited ability to distinguish true health risks or benefits attributable to playing a particular sport from preexisting differences inherent in the people who play sports at an elite level. A recent study^10^ attempted to avoid this bias by comparing career NFL players with players who only played during an NFL players’ strike. The study did not find a reduced mortality rate among career NFL players, as general population studies previously had, but instead found a nonsignificantly elevated mortality rate. However, comparison with other football players may have had limited power to detect differences in outcomes, and the study also did not have the sample size to examine specific causes of death.

To help reduce the healthy worker hire effect, we sought to compare NFL players with elite athletes of another sport, US Major League Baseball (MLB) players, as the comparison group. Although still with some potential health-related differences at the time of entry into professional sports, this comparison group should be far more comparable than the general US population. The additional need for available data, preferably with large numbers to maximize power, led us to use MLB players, on whom there is a large and comprehensive publicly available database. A direct comparison of NFL with MLB players could highlight sports factors associated with risk of diseases (eg, American-style football players have higher rates of head injuries^11^ and more cardiovascular risk factors, including excess weight, hypertension, and sleep apnea, than baseball players^12^). We specifically compared all-cause and cause-specific mortality among NFL players with that among MLB players and hypothesized that NFL players would have higher rates of all-cause mortality than MLB players. A better understanding of the long-term health consequences of American-style football may point to important risk factors and disease mechanisms and have broad public health implications.

## Methods

### Study Population

#### NFL Cohort

The NFL cohort was constructed by the National Institute for Occupational Safety and Health (NIOSH) from a 1990 NFL pension fund database as previously described.^1^ In short, it consists of 3439 NFL players who played in at least 1 season between 1959 and 1988 and had at least 5 pension-credited seasons overall. Although the NIOSH identified 20 deaths before 1979, we excluded these players from the present study to have a comparable at-risk period as MLB players, for whom our death data began in 1979, the first year of electronic National Death Index (NDI) data.^1^ Therefore, the NFL cohort for this study was composed of 3419 former players who were born between 1915 and 1965. Documentation of written consent was waived by the NIOSH institutional review board for the mortality study. The study protocol was approved by institutional review boards of the Harvard T.H. Chan School of Public Health and the National Center for Health Statistics. This cohort study followed the Strengthening the Reporting of Observational Studies in Epidemiology (STROBE) reporting guideline.

#### MLB Cohort

We identified MLB players and their career data from the publicly available Lahman Baseball Database^13^ of all MLB players who appeared in at least 1 game at the professional level in the American and National leagues, as well as earlier leagues (American Association, Union Association, Players League, Federal League, and National Association), from 1871 through 2006 (n = 16 637). Because playing career length may be related to the age at death^14,15^ and because the NFL cohort was restricted to players who played at least 5 pension-credited seasons, we excluded MLB players with fewer than 5 playing seasons (n = 9659). For additional comparability with the NFL cohort on playing era and birth cohort, we excluded MLB players whose last game was before 1959 (n = 2538) and players born before 1915 (n = 1) or after 1965 (n = 1677). Because we identified mortality via NDI linkage, the remaining players known to have died before 1979 (n = 24) or after 1979 outside of the United States and territories (n = 30) were excluded. Therefore, the MLB cohort in the present analyses was composed of 2708 former players. While decedents are not human participants and consent was thus not required, a confidentiality agreement was signed with the NDI before their release of data to us.

### Outcome Ascertainment

Causes of death for both cohorts were identified from January 1, 1979, through December 31, 2013, and were based on the *International Classification of Diseases* revision in effect at the time of death (eTable 1 in the Supplement). The dates of analysis were January 2016 to April 2019. Because certain diseases may more likely be captured as a contributing cause than an underlying cause, especially diseases with long durations,^16^ we conducted separate analyses for having the cause of death as (1) an underlying cause alone and (2) either an underlying cause or a contributing cause.

For the NFL cohort, vital status was ascertained by the NIOSH through pension fund records, the Social Security Administration, and the Internal Revenue Service.^1^ When death information was not provided by the NDI, a certified nosologist coded causes of death from death certificates obtained from state vital statistics offices.^1^ The data used for the present study included NDI follow-up that extended 6 years beyond a prior study^2^ on neurodegenerative disease mortality in this cohort.

**Figure 1.**
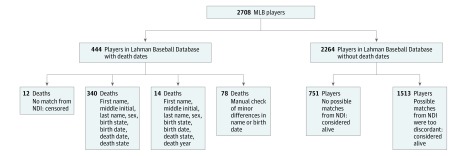
Flowchart of Matching Major League Baseball (MLB) Players With the National Death Index (NDI) Information on death date and death state was available for 444 MLB players from the Lahman Baseball Database, and NDI linkage identified 432 (97.3%) of these deaths. Of the 432 identified deaths, 340 deaths (78.7%) were exact matches, and 14 deaths (3.2%) were found after allowing nonmatch on death day and death month. We manually checked and confirmed the remaining 78 deaths (18.1%) in the Lahman Baseball Database that had minor differences in name or birth date with NDI records. The remaining 2264 of the 2708 players in the Lahman Baseball Database without death dates were considered alive.

For the MLB cohort, we used available data from the Lahman Baseball Database to match to the NDI based on name, sex, birth date, birth state, death date, and death state. A flowchart for the matching process is shown in Figure 1. Among 444 players with death dates in the Lahman Baseball Database, we successfully matched 432 to the NDI. Specific causes of death were not provided by the NDI for 1 match due to state reporting restrictions. We censored this player and 12 other players with death dates in the Lahman Baseball Database but without matches from the NDI at the day before their deaths. As a result, the final analysis consisted of 431 dead players and 2277 alive or censored players.

### Statistical Analysis

We calculated hazard ratios (HRs) from Cox proportional hazards regression models to investigate whether mortality among NFL players differed from that among MLB players in this retrospective cohort study. Because NDI electronic death data began in 1979 and we otherwise did not have causes of death data before 1979, the at-risk period for each player began on January 1, 1979 (even if the player had completed his fifth season before then), or July 1 at the end of his fifth season, whichever was later. Follow-up ended on the death date, censoring date, or last day of NDI follow-up on December 31, 2013, whichever came first. Attained age was used as the timescale (metameter) for the Cox proportional hazards regression models, in which players were followed up from the age at the start of the at-risk period to the age at death or censoring date, to flexibly account for confounding by age.^17^ These models accounted for left truncation in our data created by players starting follow-up at different ages due to our at-risk period start definition.^18^ Deaths from other causes in the cause-specific analyses were treated as censored events. We estimated excess deaths from survival curves predicted from the Cox proportional hazards regression models.

Models were adjusted for race (white vs nonwhite) and decade of birth year (1915-1924, 1925-1934, 1935-1944, 1945-1954, or 1955-1965). Information on race was available in the NFL cohort; race in the MLB cohort was confirmed by the NDI as white or nonwhite for players with death matches. For MLB players who were still alive at the end of follow-up, race was predicted using US census data based on last name, birth county, and being male for players born in the United States and Puerto Rico and based on last name only for players born elsewhere (Package wru, version 0.1-7; The R Foundation).^19,20^ Players with probability of being at least 50% white were considered to be of white race. This prediction had 85% accuracy when validated against all players in the full MLB cohort with known race from the NDI. The method also yielded percentages of white individuals that were close to reported percentages in the MLB (predicted 74% vs reported 71% in 1979 and predicted 64% vs reported 61% in 2006).^21^

All hypothesis tests were 2-sided and assessed at *P* < .05 level of significance. Analyses were conducted using R Statistical Software, version 3.3.1 (R Project for Statistical Computing). We used cox.zph (R package survival) to test the proportional hazards assumption, and global and league (NFL vs MLB) *P* values for all models were not significant.

## Results

The mean (SD) age at debut for NFL players was 21.8 (1.2) years, approximately 1.5 years younger than MLB players at debut (mean [SD] age, 23.3 [2.2] years) and similarly younger at the start of follow-up (Table 1). Sixty percent (2053 of 3419) of NFL players were of white race compared with 77.8% (2106 of 2708) of MLB players. By the end of follow-up in 2013, there were 517 deaths in 106 191 person-years among 3419 NFL players and 431 deaths in 79 828 person-years among 2708 MLB players (Table 1). The mean (SD) age at death was 59.6 (13.2) years among NFL players and 66.7 (12.3) years among MLB players, and 73.5% (380 of 517) and 58.7% (253 of 431) of deaths occurred before age 70 years among NFL and MLB players, respectively. Cardiovascular and neurodegenerative conditions, respectively, were noted as underlying or contributing causes in 498 and 39 deaths in the NFL and 225 and 16 deaths in the MLB (Table 2).

**Table 1.** Characteristics of NFL and MLB Players

Characteristic	NFL (n = 3419)	MLB (n = 2708)
Person-years of follow-up, No.	106 191	79 828
No. of deaths	517	431
White race, No. (%)	2053 (60.0)	2106 (77.8)
Age, mean (SD), y		
At debut in league	21.8 (1.2)	23.3 (2.2)
At start of follow-up	31.7 (6.5)	34.0 (8.2)
At death	59.6 (13.2)^a^	66.7 (12.3)^b^
At last date observed, alive or censored	63.2 (7.9)^c^	62.8 (10.1)^d^

Abbreviations: MLB, Major League Baseball; NFL, National Football League.

^a^ Among deaths (n = 517).

^b^ Among deaths (n = 431).

^c^ Among players alive or censored (n = 2902).

^d^ Among players alive or censored (n = 2277).

**Table 2.** Adjusted HRs for Overall and Cause-Specific Mortality Comparing NFL With MLB Players^a^

Cause of Death	Underlying Cause			Underlying or Contributing Cause^b^		
	NFL, No.	MLB, No.	HR (95% CI)	NFL, No.	MLB, No.	HR (95% CI)
All deaths	517	431	1.26 (1.10-1.44)	517	431	1.26 (1.10-1.44)
All cancers	132	150	0.91 (0.71-1.16)	171	156	1.11 (0.88-1.39)
All cardiovascular diseases	200	147	1.52 (1.21-1.90)	498	225	2.40 (2.03-2.84)
All neurodegenerative diseases	22	13	2.07 (1.01-4.23)	39	16	2.99 (1.64-5.45)
Dementia/Alzheimer disease	6	7	1.28 (0.41-4.07)	16	10	2.26 (0.99-5.17)
Amyotrophic lateral sclerosis	9	3	2.81 (0.75-10.51)	10	3	3.10 (0.84-11.38)
Parkinson disease	7	3	3.08 (0.76-12.42)	14	5	3.45 (1.21-9.83)
Suicide	11	5	1.59 (0.54-4.69)	11	5	1.59 (0.54-4.69)

Abbreviations: HR, hazard ratio; MLB, Major League Baseball; NFL, National Football League.

^a^ Cox proportional hazards regression models using age as the timescale were adjusted for race and decade of birth.

^b^ The total for any category is the number of death certificates that had a cause of death in that category indicated at least once.

**Figure 2.**
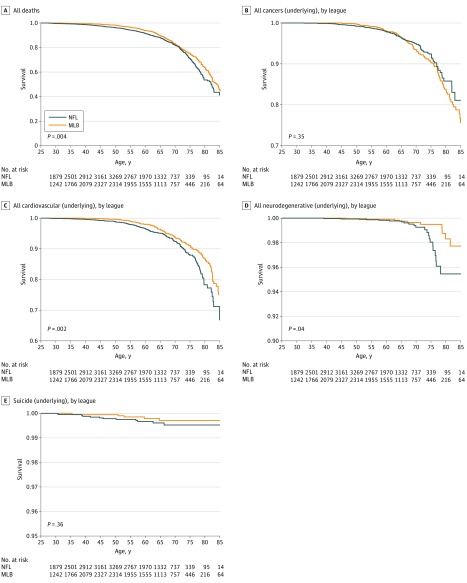
Kaplan-Meier Curves for Underlying Causes of Death by League Survival by age is shown for each league; *P* values are from log-rank tests. MLB indicates Major League Baseball; NFL, National Football League.

**Figure 3.**
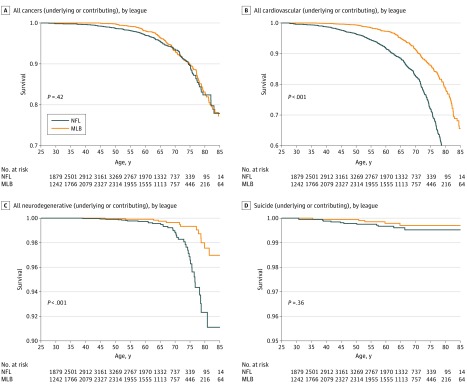
Kaplan-Meier Curves for Underlying or Contributing Causes of Death by League Survival by age is shown for each league; *P* values are from log-rank tests. MLB indicates Major League Baseball; NFL, National Football League.

The unadjusted HR for all-cause mortality comparing NFL players with MLB players was 1.21 (95% CI, 1.06-1.39); unadjusted HRs for cause-specific mortality are listed in eTable 2 in the Supplement. Kaplan-Meier curves for unadjusted all-cause and cause-specific survival by league are shown in Figure 2 and Figure 3. Compared with MLB players, NFL players had significantly elevated adjusted rates of all-cause (HR, 1.26; 95% CI, 1.10-1.44), cardiovascular disease (HR, 2.40; 95% CI, 2.03-2.84), and neurodegenerative disease (HR, 2.99; 95% CI, 1.64-5.45) mortality when considering underlying or contributing causes but no difference in rates of cancer mortality (Table 2). The NFL players had a significantly elevated mortality rate from Parkinson disease (PD), while mortality rates from dementia and/or Alzheimer disease (AD) and amyotrophic lateral sclerosis (ALS) were not significantly elevated. These results were attenuated when considering the underlying cause alone. Suicide was elevated among NFL players but was not statistically significant.

Survival curves for hypothetical populations of 1000 NFL players and 1000 MLB players predicted an excess of 4, 10, and 21 all-cause deaths among NFL players compared with MLB players by age 55, 65, and 75 years, respectively (Table 3). For cardiovascular diseases indicated as an underlying or a contributing cause, NFL players had an excess of 16 deaths by age 55 years and an excess of 77 deaths by age 75 years compared with MLB players. For neurodegenerative diseases, NFL players had an excess of 1 death by age 55 years and an excess of 11 deaths by age 75 years compared with MLB players. Of the 11 excess neurodegenerative deaths, 5 were due to PD, 3 were due to dementia and/or AD, and 2 were due to ALS (the difference in total is because of rounding).

**Table 3.** Estimated Number of Deaths in Hypothetical Populations of 1000 NFL and 1000 MLB Players by Attained Age

Cause of Death (Underlying or Contributing)	Attained Age, y	Estimated No. (95% CI) of Deaths	
		NFL	MLB
All causes	55	52 (46-60)	48 (43-54)
	65	122 (110-134)	112 (103-122)
	75	291 (266-317)	270 (251-290)
All cardiovascular diseases	55	51 (44-58)	35 (31-41)
	65	114 (103-127)	81 (73-90)
	75	282 (257-309)	205 (188-225)
All neurodegenerative diseases	55	2 (1-5)	1 (1-3)
	65	5 (3-9)	3 (2-5)
	75	28 (18-42)	17 (12-26)

Abbreviations: MLB, Major League Baseball; NFL, National Football League.

## Discussion

In this retrospective cohort study, we found a large and significantly elevated rate of all-cause mortality, and specifically cardiovascular and neurodegenerative mortality, among NFL players compared with MLB players. The absolute expected excess number of cardiovascular deaths among NFL players compared with MLB players was somewhat high because deaths with these conditions were common. In contrast, neurodegenerative disease–related deaths were less common. Despite a large relative increase in neurodegenerative mortality among NFL players, the absolute number of these deaths and the excess number of deaths among NFL players were still small.

The prior study^2^ of the same NFL cohort found lower all-cause and cardiovascular mortality compared with the general US population. This likely reflects healthy worker hire effects in that one must be healthier than people of the same age in the general US population to get into the NFL.^8,9^ In addition, aspects of playing in the NFL (eg, exercise regimens) may confer health benefits compared with the general population.^22^ In contrast, compared with another group of elite team sport athletes (MLB players), who would be much more comparable in not only levels of fitness and health but also many other characteristics of playing a professional sport, our results suggest that NFL players have a greater risk of all-cause and cardiovascular mortality. Our all-cause mortality result was opposite to the conclusion of the only other study^23^ of which we are aware that compared football players with baseball players. However, in that study, the MLB group was started almost 50 years before the NFL group, resulting in MLB players of earlier birth cohorts. This was not accounted for in the combined sport analysis, which would have skewed the results toward greater mortality among the MLB players.

The prior study^2^ of NFL players found higher neurodegenerative mortality among NFL players compared with the general US population, similar to our findings comparing MLB players. This suggests that the health advantages at the time of getting into and playing in the NFL that confer lower all-cause and cardiovascular mortality compared with the general population are not related to later neurodegenerative disease risk. Lower suicide mortality has also been reported among NFL players compared with the general population.^24^ We did not find this when comparing NFL players with MLB players; in fact, the point estimate was elevated but not statistically significant. Suicide among NFL players has been more commonly reported in recent years,^24^ so this merits monitoring over time because it could be changing.

Another recent study^10^ that compared career NFL players with NFL replacement players who played during a strike found an increased rate of overall mortality among the career players (HR, 1.38; 95% CI, 0.95-1.99). Although not statistically significant, the effect estimate was comparable to our findings (HR, 1.26). The lack of significance was likely related to fewer players and thus lower power. Furthermore, NFL replacement players presumably had football-related exposures that were at least similar to those of career NFL players, and this could have muted the differences in football-related exposures that predispose to earlier mortality. Assuming that MLB players would have had much less football-related exposure in high school and college (despite many athletes likely playing dual sports at younger ages) than either career or replacement NFL players, the similarity in the findings of our study and the above NFL replacement player study may suggest that exposures associated specifically with playing in the NFL are most relevant for the increased mortality. However, without comprehensive data on earlier playing experience, this cannot be definitively concluded.

The increased cardiovascular disease mortality rate we observed could be related to differences between NFL and MLB players in physiological factors relevant to cardiovascular health.^12,25^ In the Football Players Health Study,^26,27^ anterior cruciate ligament tear and early-career weight gain (both likely more common or more pronounced in NFL players than MLB players) were found to be associated with increased cardiovascular and cardiometabolic disease. Differences in tobacco use habits are also a possibility. However, although smokeless tobacco use is particularly prevalent in baseball^28,29,30^ and its use increases the risk of cardiovascular outcomes,^31^ we still saw higher rates of cardiovascular disease mortality in NFL players. This might suggest that our findings are an underestimate of cardiovascular death differences between NFL and MLB players that are not associated with smokeless tobacco use. A higher rate of cigarette smoking (a strong risk factor for cardiovascular diseases) in NFL players could in theory underlie the increased rate of cardiovascular deaths, but it is not clear that NFL players smoke more than MLB players. Both leagues have had a close association with the cigarette industry,^32^ and a study^33^ among collegiate players suggested similar smoking prevalence, possibly higher among baseball players. In addition, although smoking can lead to various cancers, we did not see differences in rates of all cancers.

Historically, several studies have suggested a possible association between head injury and dementia and AD,^34,35^ PD,^36,37,38,39^ and ALS,^40,41,42^ although some studies^43,44^ did not observe such associations. In the prior study^2^ of NFL players, both dementia and/or AD mortality and ALS mortality were significantly elevated by about 4-fold over the general population, while PD mortality was not significantly elevated. For ALS specifically, the potential that some athletes were at an increased risk garnered more attention after reports of significantly higher ALS mortality among Italian professional soccer players and among NFL players.^45,46,47,48,49^ However, comparisons of other elite athletes (cyclists and basketball players) with general populations did not find elevated ALS mortality.^46^

A limitation of the studies of neurodegenerative disease is that professional athletes were compared with general populations. The elevated neurodegenerative disease mortality rate we found provides some of the strongest evidence to date that factors common across elite athletes of different sports, such as physical activity,^50^ are unlikely to account for increased neurodegenerative disease risk seen specifically among American-style football players. Instead, sports factors are more likely the causal factors underlying this difference in risk. For instance, head trauma and repetitive head injuries, as well as many subconcussive blows, are more frequently reported in American-style football and soccer than in baseball, cycling, and basketball.^11,46^ Therefore, the difference in head injuries across sports could account for their differences in neurodegenerative disease risk. Because of the period of our study, few (if any) deaths were likely to have been attributed to chronic traumatic encephalopathy.

Body composition could also account for some differences in disease risk. American-style football players generally have higher body mass indexes, an indirect measure of body composition, than baseball players, possibly underlying the elevated cardiovascular disease mortality in our results. In addition, the Football Players Health Study^26^ found that early-career weight gain was associated with worse neurocognitive health in later life. However, this difference would not likely explain an elevated rate specifically of ALS mortality in NFL players because higher body mass index has been reported to be protective for ALS risk^51^ and could contribute to why the elevated rate we found was not statistically significant. Also, the body mass indexes of Italian soccer players likely differ more from those of American-style football players than players of other sports, yet both soccer players and American-style football players appear to have higher ALS risk than other athletes.

### Limitations and Strengths

Several limitations should be considered in the interpretation of our results. Race was imputed for some MLB players, but our estimates had good concordance with known race in our cohort. We also did not have information on other factors that may contribute to neurological and other conditions, such as genetics, family history, or lifestyle and environmental risk factors. Whether our results among NFL players apply to athletes of other sports is unclear; studies of other athletes are needed. Our results may also be limited to NFL players in the playing years considered because there have been changes in sports characteristics over time, such as helmet use, training regimen, and smoking prevalence. The use of MLB players as a comparison group for NFL players has the advantage over comparisons with general populations of minimizing healthy worker hire effect biases and thus better identifying mortality differences among NFL players that are not just a function of generally better fitness and other factors related to making it into a professional sports league.^9^ However, there could still be other differences between NFL and MLB players that are not specific to the actual play of NFL football and could account for mortality differences we observed. Ultimately, comparisons among NFL players by differences in specific aspects of NFL play would be needed to identify specific factors that could lead to differences in mortality risks for different outcomes. In addition, our study is also the first study to date, to our knowledge, to directly compare neurodegenerative diseases among athletes of different sports.

## Conclusions

The results of this study found that NFL players had a significantly elevated rate of all-cause mortality compared with MLB players, driven by elevated rates of cardiovascular and neurodegenerative mortality. Our results suggest that some exposures more associated with playing professional American-style football than baseball are associated with an increased risk of cardiovascular and neurodegenerative disease mortality. This indicates the need for cohort studies of football players with more detailed information on specific aspects of players’ football experience to isolate what specific factors are associated with increased cardiovascular and neurodegenerative risk, which could provide more insight into potentially modifiable factors that might mitigate some of the excess mortality rate we found among NFL players. Our findings also highlight the need for complementary pathophysiological studies of former NFL athletes to delineate the biological basis for the findings we observed herein. Such efforts could lead to better general understanding of the pathophysiology of these conditions and suggest interventions that could reduce the burden of these outcomes among American-style football players.

## Notes

Supplement. **eTable 1.** International Classification of Diseases Codes for Causes of Death **eTable 2.** Unadjusted Hazard Ratios (95% CIs) for Overall and Cause-Specific Mortality Comparing NFL to MLB Players
