# Supplementary material for: Mortality Among Professional American-Style Football Players and Professional American Baseball Players
Source: JAMA Netw Open. 2019 May 24;2(5):e194223. doi: 10.1001/jamanetworkopen.2019.4223 (PMC6632140; doi:10.1001/jamanetworkopen.2019.4223)
Supplement: Supplement. — eTable 1. International Classification of Diseases Codes for Causes of Death eTable 2. Unadjusted Hazard Ratios (95% CIs) for Overall and Cause-Specific Mortality Comparing NFL to MLB Players [file jamanetwopen-2-e194223-s001.pdf]

Supplementary Online Content

Nguyen VT, Zafonte RD, Chen JT, et al. Mortality among professional American-style football players and professional American baseball players. *JAMA Netw Open*. 2019;2(5):e194223. doi:10.1001/jamanetworkopen.2019.4223

eTable 1. International Classification of Diseases Codes for Causes of Death

eTable 2. Unadjusted Hazard Ratios (95% CIs) for Overall and Cause-Specific Mortality Comparing NFL to MLB Players

This supplementary material has been provided by the authors to give readers additional information about their work.

© 2019 Nguyen VT et al. *JAMA Network Open*.

eTable 1. International Classification of Diseases Codes for Causes of Death

Cause of death	ICD-9 Codes	ICD-10 Codes
All cancers	140-208, 273.3	C00-C97
All cardiovascular diseases	390-459	G45, I00-I99, M30-M31, R00, R58
Dementia/Alzheimer’s disease	290.0-290.3, 331.0	G30
Amyotrophic lateral sclerosis	335.2	G12.2
Parkinson’s disease	332	G20-G21
Suicide	E950-E959	X60-X84, Y87.0

Abbreviations: ICD, International Classification of Diseases.

© 2019 Nguyen VT et al. *JAMA Network Open*.

eTable 2. Unadjusted[a] Hazard Ratios (95% CIs) for Overall and Cause-Specific Mortality Comparing NFL to MLB Players

Cause of Death	Underlying Cause HR (95% CI)	Underlying or Contributing Cause[b] HR (95% CI)
All deaths	1.21 (1.07 to 1.39)	1.21 (1.07 to 1.39)
All cancers	0.89 (0.70 to 1.13)	1.10 (0.88 to 1.37)
All cardiovascular diseases	1.41 (1.13 to 1.75)	2.28 (1.94 to 2.68)
All neurodegenerative diseases	2.09 (1.03 to 4.21)	2.99 (1.65 to 5.43)
Dementia/Alzheimer's disease	1.32 (0.43 to 4.04)	2.16 (0.96 to 4.87)
Amyotrophic lateral sclerosis	2.56 (0.69 to 9.54)	2.84 (0.78 to 10.41)
Parkinson's disease	3.16 (0.80 to 12.55)	3.77 (1.34 to 10.65)
Suicide	1.63 (0.57 to 4.70)	1.63 (0.57 to 4.70)

Abbreviations: NFL, National Football League; MLB, Major League Baseball; HR, hazard ratio; CI, confidence interval.
[a]Age was accounted for as the timescale of the Cox model, but race and decade of birth were not included in the models.
[b]The total for any category is the number of death certificates that had a cause of death in that category indicated at least once.

© 2019 Nguyen VT et al. *JAMA Network Open*.
